# Concomitant Use of Psychotropic Medication With Stimulants for the Treatment of ADHD in Children and Adolescents: A Retrospective Insurance Claims Study in the United States

**DOI:** 10.1177/1087054718784668

**Published:** 2018-07-10

**Authors:** Zhou Zhou, Keith A. Betts, Iryna Bocharova, David Kinrich, William M. Spalding

**Affiliations:** 1 Analysis Group, Inc., Boston, MA, USA; 2 Shire Outcomes Research & Epidemiology, Lexington, MA, USA

**Keywords:** ADHD, concomitant treatment, stimulants

## Abstract

**Objective:** To evaluate annual concomitant psychotropic medication use among stimulant-treated children/adolescents with ADHD. **Method:** Children/adolescents with ≥1 primary ADHD diagnosis who had received ≥30 days of stimulant medication were identified from insurance claims for each calendar year (2011-2014). Use of 15 psychotropic medications concomitantly with stimulants was evaluated and their prevalence in each year was calculated overall and by medication category for children (6-12 years) and adolescents (13-17 years). **Results:** Each year 133,354 to 157,303 children and 95,632 to 111,280 adolescents were included. Annual period prevalence of any concomitant psychotropic medication use was 22.9% to 25.0% for children and 25.2% to 28.2% for adolescents. The most common medication categories included selective serotonin reuptake inhibitors (children: 6.8%-7.9%; adolescents: 12.7%-14.9%), atypical antipsychotics (4.2%-5.4%; 5.3%-6.3%), and guanfacine extended release (5.1%-7.0%; 2.3%-3.6%). **Conclusion:** Around a quarter of children/adolescents with ADHD were prescribed psychotropic medication concomitant to stimulant treatment, although only 2 of the 15 medication classes studied were Food and Drug Administration (FDA)-approved for adjunctive use.

## Introduction

ADHD is one of the most commonly diagnosed neurobehavioral disorders of childhood and adolescence (Akinbami, Liu, Pastor, & Reuben, 2011; Constantine & Tandon, 2008; Fulton et al., 2009; Pastor & Reuben, 2008). The worldwide prevalence of ADHD among children and adolescents ranges from 5.9% to 7.1% (Thomas, Sanders, Doust, Beller, & Glasziou, 2015; Willcutt, 2012), and the economic burden of the disease is substantial (Doshi et al., 2012).

Stimulants are effective first-line pharmacological treatments for children and adolescents with ADHD (Strange, 2008; Wolraich et al., 2011); however, a subset of patients with ADHD requires supplementation of their existing stimulant regimen with additional medications due to inadequate/partial response or dose-limiting side effects (Banaschewski, Roessner, Dittmann, Santosh, & Rothenberger, 2004; Olfson, 2004; Pliszka, 2003). At the time of this study, the only medications approved by the U.S. Food and Drug Administration (FDA) for adjunctive therapy in stimulant-treated children and adolescents with ADHD were guanfacine extended release (XR) and clonidine XR (Childress, 2012; Ming, Mulvey, Mohanty, & Patel, 2011).

Despite guidance from the American Academy of Pediatrics against off-label adjunctive drug therapy for the treatment of ADHD (Wolraich et al., 2011), off-label concomitant use of psychotropics is common in the clinical setting (Cooper et al., 2006; Cooper, Hickson, Fuchs, Arbogast, & Ray, 2004; Czaja & Valuck, 2012; Jensen et al., 2005; Pappadopulos et al., 2002; Spencer & Biederman, 2002; Zito et al., 2008). In general, efficacy and safety of off-label use of concomitant psychotropic medication have not been thoroughly investigated, and the subsequent risk–benefit profiles of drug regimens used in practice are insufficiently described (Bussing & Winterstein, 2012).

Several studies have reported that ADHD is the leading diagnosis associated with prescriptions of atypical antipsychotics (AAPs) even though AAPs are not indicated for this condition (Cooper et al., 2006; Matone et al., 2012; Pathak, West, Martin, Helm, & Henderson, 2010; Sikirica et al., 2012b; Sikirica et al., 2014; Weiss et al., 2009). Studies have suggested potential risks of polypharmacy with AAPs, including induction of metabolic syndrome (Bussing & Winterstein, 2012; Findling, Steiner, & Weller, 2005; Maglione et al., 2011; Penzner et al., 2009; Weiss et al., 2009; Yanofski, 2010). Furthermore, abrupt discontinuation of stimulants or AAPs when used concomitantly to treat ADHD may lead to dyskinesias (Yanofski, 2010).

A previous study estimated that the 1-year prevalences of concomitant psychotropic medication use with stimulants (in 2009) among children and adolescents with ADHD were 20.3% and 23.4%, respectively (Betts et al., 2014). Since then, important treatment and formulary changes have created additional treatment options for children and adolescents with ADHD, particularly, those who require augmentation of their stimulant regimen. In particular, the FDA has approved the nonstimulants guanfacine XR (as monotherapy in 2009 and as an adjunct to stimulant therapy in early 2011) and clonidine XR (as monotherapy and an adjunct to stimulant therapy in late 2010; Thomas, 2013).

The current study examined trends in concomitant psychotropic medication use (including off-label use) among stimulant-treated children and adolescents with ADHD from 2011 to 2014. As rates of ADHD diagnosis and treatment are known to vary by age category (Constantine & Tandon, 2008; Cooper et al., 2004), children (aged 6-12) and adolescents (aged 13-17 years) were analyzed separately. Children and adolescents with ADHD commonly exhibit additional psychiatric and neurologic comorbidities, and many of the medication categories that are used off-label for ADHD may also be used for the treatment of such comorbidities among patients with ADHD (Larson, Russ, Kahn, & Halfon, 2011). Thus, in addition to examining trends in the overall population, the prevalence of concomitant psychotropic medication use was also estimated among children and adolescents without psychiatric and neurologic comorbidities.

## Method

### Data

This study was conducted using data from the Truven Health MarketScan Commercial Claims and Encounters (MarketScan^®^) database from January 1, 2011 to December 31, 2014. These data include commercial health insurance claims (inpatient and outpatient medical, and outpatient pharmacy) and enrollment information from large US employers and health insurance plans. Such plans provide private health care coverage for more than 50 million employees, their spouses, and dependents and reflect a variety of fee-for-service, preferred provider organization, and capitated health plans (i.e., a health plan that allows payment of a flat fee for each patient it covers).

As the study was a retrospective analysis of de-identified administrative claims data, it was fully compliant with the Health Insurance Portability and Accountability Act and, thus, exempt from institutional review board approval.

### Sample Selection

The study population included patients with at least one primary ADHD diagnosis (International Classification of Diseases, 9th Revision, Clinical Modification [ICD-9-CM] codes 314.00 or 314.01; Centers for Disease Control and Prevention, 2017) between January 1, 2011 and December 31, 2014. For each calendar year between 2011 and 2014, patients were required to be aged 6 to 17 years as of January 1, have continuous health plan enrollment eligibility from January 1 to December 31, and have at least a 30-day stimulant medication course (identified using generic product identifier codes) between January 1 and December 31. The stimulant course could consist of one or more prescription fills if the time on treatment was at least 30 days. Noncontinuous interrupted treatment periods of up to 30 days were allowed. The first stimulant filled in each respective year was defined as the index stimulant for patients in that year.

A subpopulation of patients with ADHD who did not have any other diagnosed psychiatric or neurologic comorbidities was also identified (“patients with noncomorbid ADHD”); this consisted of patients who did not have any primary diagnoses associated with a psychiatric or neurological condition, including bipolar disorder, dementia, mania, schizophrenia, tics, adjustment reaction, anxiety disorder, conduct disorder, depression, insomnia, learning disability, obsessive compulsive disorder, oppositional defiant disorder, substance abuse, pervasive developmental disorder, epilepsy, and other psychiatric and neurological disorders (as identified by ICD-9-CM codes; see Appendix).

### Measures

During each calendar year, concomitant use of 15 distinct categories of medication was evaluated. These medications either had an FDA-approved indication for ADHD or were used off-label to treat ADHD in practice (Cooper et al., 2006; Cooper et al., 2004; Kreider et al., 2014; Spencer & Biederman, 2002). The 15 categories were two classes of short-duration-of-action stimulants (amphetamine [AMPH] short acting [SA] and methylphenidate [MPH] SA); two classes of long-duration-of-action stimulants (AMPH long acting [LA] and MPH LA); three nonstimulants approved for ADHD (atomoxetine, clonidine XR, and guanfacine XR); two nonstimulants not approved for ADHD (clonidine immediate release [IR] and guanfacine IR); two classes of antipsychotics (AAPs and typical antipsychotics [TAPs]); and four classes of antidepressants (bupropion, selective serotonin reuptake inhibitors [SSRIs], serotonin-norepinephrine reuptake inhibitors (SNRIs), and tricyclic antidepressants [TCAs]).

For a medication fill to qualify as concomitant usage, the medication had to have been filled within the same calendar year as the index stimulant and have at least 30 days of supply overlap with the index stimulant, and there had to be at least one primary diagnosis of ADHD prior to the end of medication overlap. For a stimulant to qualify as concomitant usage, it had to be a different class to the index stimulant. Patients were classified into multiple categories if they used medications from more than one category concomitantly (patients using medications from multiple different categories concomitantly would be counted multiple times, whereas patients who had separate periods of concomitant use within the same category were only counted once). When evaluating multiple concomitant-use events, the periods of overlap between the index stimulant and differing concomitantly used agents were not required to be mutually exclusive.

### Analyses

The 1-year period prevalence of concomitant psychotropic medication use, overall and by medication category, was calculated for each calendar year (2011-2014). The overall 1-year prevalence was calculated as the percentage of patients experiencing at least one concomitant-use event with any of the 15 distinct medication classes. The 1-year period prevalence by medication category was calculated as the proportion of patients experiencing at least one concomitant-use event with the given medication category per calendar year. As a sensitivity analysis, the 1-year period prevalence of concomitant psychotropic medication use, overall and by medication category, was calculated among the subpopulation of patients with noncomorbid ADHD. Results are reported in the text as ranges for the four 1-year periods.

Separate analyses were performed for the child and adolescent subpopulations, and all analyses were performed using SAS Version 9.3 (SAS Institute, Cary, NC).

## Results

### Children

**Figure 1.**
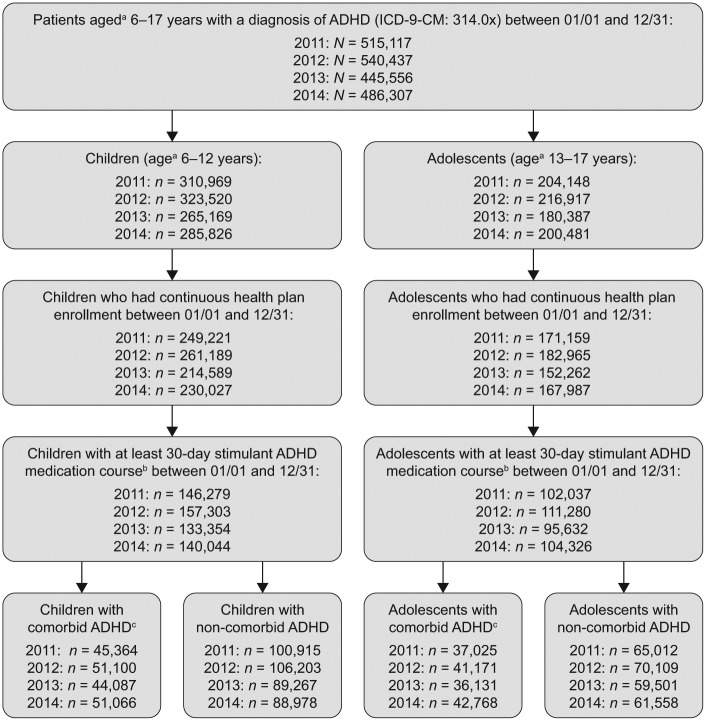
Sample selection flowchart. ^a^Patients’ age on January 1 of respective year. ^b^The stimulant course could consist of one or more prescription fills as long as the time on the treatment was at least 30 days. Noncontinuous treatment interruption periods up to 30 days were allowed. ^c^Psychiatric and neurologic comorbidities included bipolar disorder, dementia, mania, schizophrenia, tics, adjustment disorders, anxiety disorder, conduct disorder, depression, insomnia, learning disability, obsessive compulsive disorder, oppositional defiant disorder, substance abuse, pervasive developmental disorder, epilepsy, and other psychiatric and neurological disorders.

Overall, between 133,354 and 157,303 children met the inclusion criteria for each year of the analysis (Figure 1). The mean age in each study year ranged from 9.31 to 9.40 years, and 71.4% to 72.1% of patients were males. Around one third of children (range: 31.0%-36.5%) had at least one diagnosed psychiatric or neurologic comorbidity. The remaining two thirds (range: 63.5%-69.0%) were considered as the subpopulation of children with noncomorbid ADHD.

Among the overall population of children, 22.9% to 25.0% added at least one other psychotropic medication to their stimulant medication during the study period, and 6.9% to 7.8% added two or more medications (Table 1). The most common concomitant psychotropic medications added were SSRIs (6.8%-7.9%), guanfacine XR (5.1%-7.0%), clonidine IR (4.9%-5.1%), AAPs (4.2%-5.4%), guanfacine IR (2.0%-3.8%), MPH SA (1.4%-1.7%), atomoxetine (1.4%-1.7%), AMPH SA (1.2%-1.5%), and AMPH LA (1.1%-1.2%; Figure 2A). Over the time period from 2011 to 2014, the prevalence of concomitant SSRIs and guanfacine XR use increased overall, whereas that of AAPs decreased. The concomitant use of guanfacine IR also increased in each year of analysis. The least frequently added (≤1%) concomitant psychotropic medications were MPH LA, clonidine XR, TCAs, bupropion, SNRIs, and TAPs (Figure 2A).

**Table 1.** Distribution of Number of Concomitant Psychotropic Medications Used per Patient.

Number	Children with ADHD (age 6-12 years)							
	Overall				Subpopulation of children with noncomorbid ADHD			
	2011	2012	2013	2014	2011	2012	2013	2014
	(*n* = 146,279)	(*n* = 157,303)	(*n* = 133,354)	(*n* = 140,044)	(*n* = 100,915)	(*n* = 106,203)	(*n* = 89,267)	(*n* = 88,978)
0	112,754 (77.08%)	118,345 (75.23%)	99,993 (74.98%)	105,284 (75.18%)	85,424 (84.65%)	88,430 (83.27%)	74,547 (83.51%)	75,937 (85.34%)
1	23,491 (16.06%)	27,330 (17.37%)	23,418 (17.56%)	23,852 (17.03%)	12,173 (12.06%)	14,051 (13.23%)	11,838 (13.26%)	10,329 (11.61%)
2	7,333 (5.01%)	8,537 (5.43%)	7,280 (5.46%)	7,939 (5.67%)	2,617 (2.59%)	2,991 (2.82%)	2,330 (2.61%)	2,201 (2.47%)
3	2,085 (1.43%)	2,464 (1.57%)	2,060 (1.54%)	2,301 (1.64%)	572 (0.57%)	615 (0.58%)	440 (0.49%)	427 (0.48%)
4	485 (0.33%)	488 (0.31%)	497 (0.37%)	545 (0.39%)	99 (0.10%)	86 (0.08%)	94 (0.11%)	71 (0.08%)
≥5	131 (0.09%)	139 (0.09%)	106 (0.08%)	123 (0.09%)	30 (0.03%)	30 (0.03%)	18 (0.02%)	13 (0.01%)
Number	Adolescents with ADHD (age 13-17 years)							
	Overall				Subpopulation of adolescents with noncomorbid ADHD			
	2011	2012	2013	2014	2011	2012	2013	2014
	(*n* = 102,037)	(*n* = 111,280)	(*n* = 95,632)	(*n* = 104,326)	(*n* = 65,012)	(*n* = 70,109)	(*n* = 59,501)	(*n* = 61,558)
0	76,309 (74.79%)	82,011 (73.70%)	69,335 (72.50%)	74,924 (71.82%)	56,089 (86.27%)	59,930 (85.48%)	50,782 (85.35%)	53,437 (86.81%)
1	18,300 (17.93%)	20,853 (18.74%)	18,692 (19.55%)	20,581 (19.73%)	7,178 (11.04%)	8,219 (11.72%)	7,073 (11.89%)	6,601 (10.72%)
2	5,597 (5.49%)	6,335 (5.69%)	5,732 (5.99%)	6,560 (6.29%)	1,409 (2.17%)	1,593 (2.27%)	1,326 (2.23%)	1,242 (2.02%)
3	1,473 (1.44%)	1,689 (1.52%)	1,504 (1.57%)	1,819 (1.74%)	267 (0.41%)	303 (0.43%)	276 (0.46%)	237 (0.39%)
4	294 (0.29%)	336 (0.30%)	313 (0.33%)	363 (0.35%)	60 (0.09%)	56 (0.08%)	35 (0.06%)	38 (0.06%)
≥5	64 (0.06%)	56 (0.05%)	56 (0.06%)	79 (0.08%)	9 (0.01%)	8 (0.01%)	9 (0.02%)	3 (<0.01%)

*Note.* Number (*n*) and frequency (%) are given for each category. Number of concomitant psychotropic medications refers to the number of different medication categories the patient used concomitantly at any time during each year. Patients are counted only once per column; therefore, rows are mutually exclusive of each other.

**Figure 2A.**
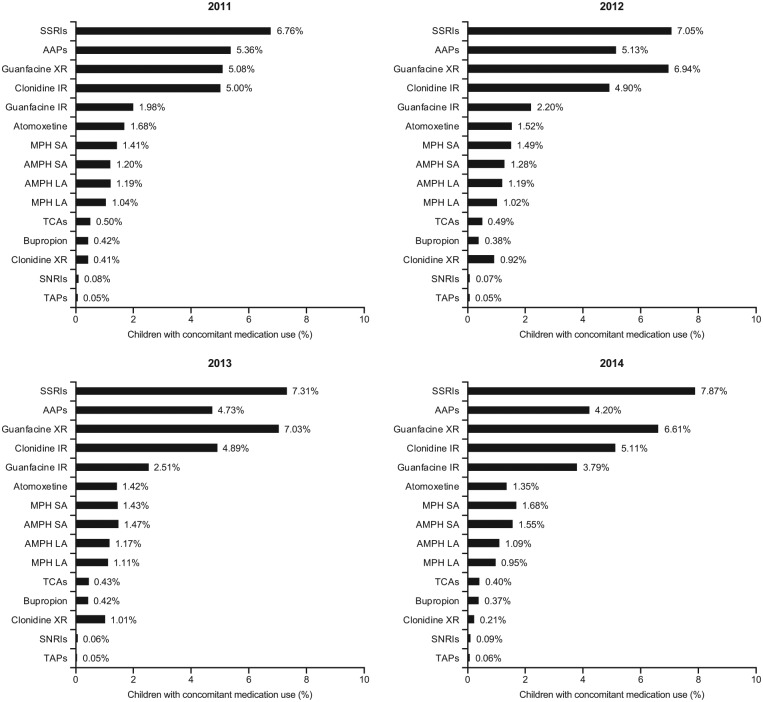
Prevalence of concomitant medication use in stimulant-treated children (6-12 years) with ADHD, by concomitant medication category.

In the subpopulation of children with noncomorbid ADHD, 14.7% to 16.7% were prescribed at least one other psychotropic medication in addition to their stimulant medication (Table 1). The most frequently added concomitant psychotropic medications were guanfacine XR (3.7%-5.3%), clonidine IR (3.1%-3.7%), SSRIs (1.6%-2.4%), AAPs (1.3%-2.3%), guanfacine IR (1.2%-2.2%), MPH SA (1.3%-1.6%), AMPH SA (1.0%-1.3%), and atomoxetine (0.8%-1.3%; Figure 2B). The least frequently added (≤1%) concomitant psychotropic medications were AMPH LA, MPH LA, TCAs, bupropion, clonidine XR, SNRIs, and TAPs (Figure 2B).

**Figure 2B.**
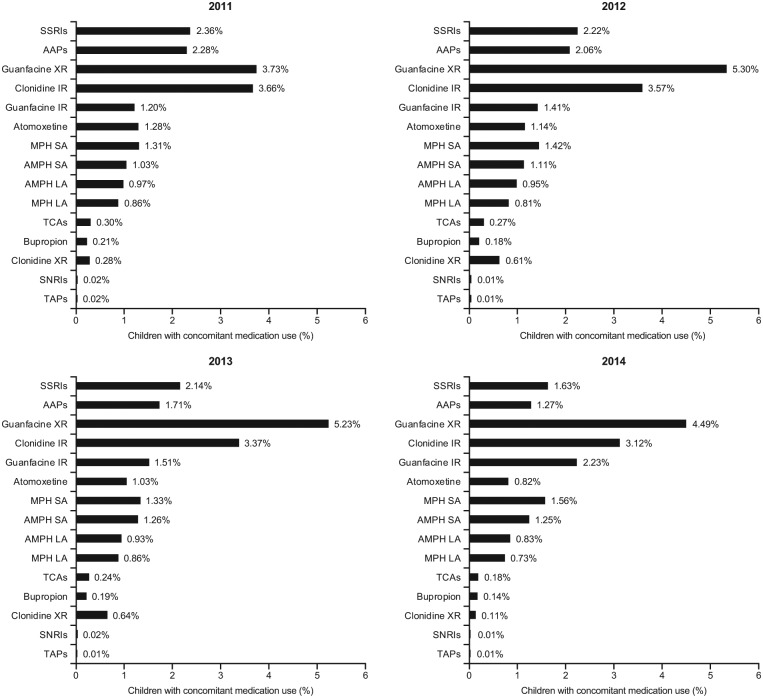
Prevalence of concomitant medication use in stimulant-treated children (6-12 years) with ADHD and without psychiatric and neurologic comorbidities, by concomitant medication category. *Note*. Medication categories are not mutually exclusive; patients were considered to have augmented multiple times if they met the concomitant use criteria for more than one medication category. SSRIs = selective serotonin reuptake inhibitors; AAPs = atypical antipsychotics; XR = extended release; IR = immediate release; MPH = methylphenidate; SA = short acting; AMPH = amphetamine; LA = long acting; TCAs = tricyclic antidepressants; SNRIs = serotonin-norepinephrine reuptake inhibitors; TAPs = typical antipsychotics.

### Adolescents

Overall, between 95,632 and 111,280 adolescents met the inclusion criteria for each year of the analysis (Figure 1). The mean age in each study year was approximately 14.90 years, and 66.9% to 67.8% of patients were males. Among this population, 36.3% to 41.0% of adolescents had at least one diagnosed psychiatric or neurologic comorbidity. The remaining adolescents (range: 59.0%-63.7%) were considered as the subpopulation of adolescents with noncomorbid ADHD.

Of the overall population of adolescents, approximately one quarter (25.2%-28.2%) added at least one other psychotropic medication to their stimulant medication during the study period and between 7.3% and 8.5% added two or more medications (Table 1). The most common concomitant psychotropic medications added were SSRIs (12.7%-14.9%), AAPs (5.3%-6.3%), guanfacine XR (2.3%-3.6%), clonidine IR (2.9%-3.2%), AMPH SA (1.5%-2.3%), and bupropion (1.8%-2.0%; Figure 3A). Over the time period from 2011 to 2014, the prevalence of concomitant SSRIs and guanfacine XR use increased overall, whereas the concomitant use of AAPs decreased. The least frequently added (≤2%) concomitant psychotropic medications were atomoxetine, guanfacine IR, MPH SA, AMPH LA, SNRIs, TCAs, MPH LA, clonidine XR, and TAPs (Figure 3A).

**Figure 3A.**
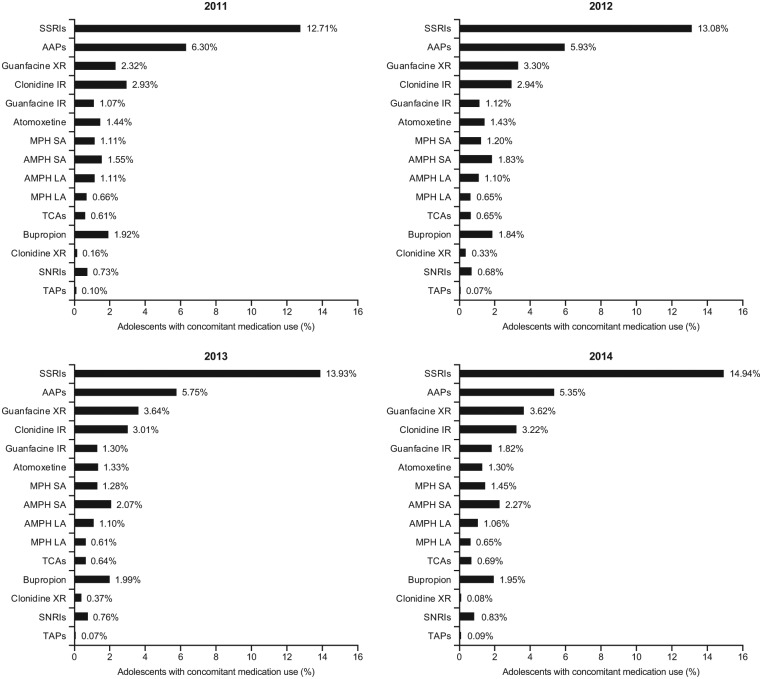
Prevalence of concomitant medication use in stimulant-treated adolescents (13-17 years) with ADHD, by concomitant medication category.

In the subpopulation of adolescents with noncomorbid ADHD, 13.2% to 14.7% were using at least one augmenting drug (Table 1). The most frequently added concomitant psychotropic medications were SSRIs (3.2%-4.3%), guanfacine XR (1.6%-2.6%), clonidine IR (1.9%-2.1%), AAPs (1.3%-2.1%), AMPH SA (1.4%-2.0%), MPH SA (1.0%-1.4%), and atomoxetine (0.9%-1.2%; Figure 3B). The least frequently added (≤1%) concomitant psychotropic medications in the population of patients with noncomorbid ADHD were AMPH LA, guanfacine IR, bupropion, MPH LA, TCAs, clonidine XR, SNRIs, and TAPs (Figure 3B).

**Figure 3B.**
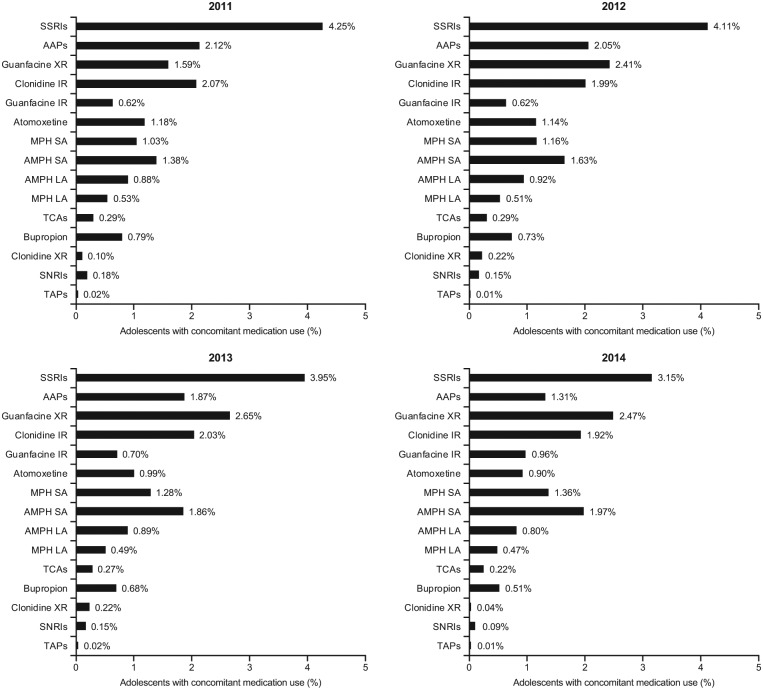
Prevalence of concomitant medication use in stimulant-treated adolescents (13-17 years) with ADHD and without psychiatric and neurologic comorbidities, by concomitant medication category. *Note*. Medication categories are not mutually exclusive; patients were considered to have augmented multiple times if they met the concomitant use criteria for more than one medication category. SSRIs = selective serotonin reuptake inhibitors; AAPs = atypical antipsychotics; XR = extended release; IR = immediate release; MPH = methylphenidate; SA = short acting; AMPH = amphetamine; LA = long acting; TCA = tricyclic antidepressants; SNRIs = serotonin-norepinephrine reuptake inhibitors; TAPs = typical antipsychotics.

## Discussion

In this large health care claims database analysis, around a quarter of children and adolescents with ADHD were prescribed at least one other category of medication concomitant to their index stimulant treatment between 2011 and 2014. Furthermore, data showed that 6.9% to 7.8% of children and 7.3% to 8.5% of adolescents were prescribed two or more categories of concomitant medication during this period. Overall, the rates of children and adolescents with ADHD being prescribed concomitant drugs increased in each year from 2011 to 2014. Reasons for this increase were not investigated, but possible reasons include an increase in the number of ADHD treatment options available or changes in prescriber attitudes.

The two most commonly used concomitant agents were SSRIs and AAPs, neither of which is approved by the FDA for the treatment of ADHD. This finding is noteworthy given the sparse clinical evidence regarding the treatment of ADHD with SSRIs and AAPs, and the potential risk of serious adverse events with AAPs (Bussing & Winterstein, 2012; Penzner et al., 2009; Yanofski, 2010). A notable rise in the use of SSRIs in both children and adolescents was observed; however, the use of AAPs decreased annually in both children and adolescents and was surpassed by the use of guanfacine XR and clonidine IR for children with ADHD.

Concomitant use of guanfacine XR increased annually from 2011 to 2014 in children (5.1%-6.6%) and adolescents (2.3%-3.6%). Furthermore, even after guanfacine XR was approved for use as adjunctive therapy to stimulants (in February 2011), concomitant use of guanfacine IR with stimulant medication increased annually from 2011 to 2014 in children (2.0%-3.8%) and adolescents (1.1%-1.8%). This was despite the lack of efficacy and safety data from well-controlled studies of guanfacine IR for ADHD, and the finding that guanfacine XR is a cost-effective alternative to stimulant monotherapy among children and adolescents with suboptimal response to stimulants (Sikirica et al., 2012a).

Although there was some variation over the entire period in both children and adolescents, an increase in use of clonidine IR was observed, and the off-label formulation clonidine IR was utilized more than the on-label formulation clonidine XR.

Children and adolescents with ADHD commonly exhibit additional psychiatric and neurologic comorbidities (Larson et al., 2011). As such, patients with ADHD-related comorbidities who were categorized as patients using multiple concomitant psychotropics may have been concurrently using the additional (nonindex) drug to treat comorbidities and the stimulant to treat ADHD (e.g., patients with ADHD with comorbid depression who use concomitant SSRIs). To address these concerns, a sensitivity analysis was conducted among a subpopulation of children and adolescents who did not have any psychiatric or neurologic comorbidities. Among these children and adolescents with noncomorbid ADHD, the prevalence of concomitant psychotropic medication use was lower than that in the overall population, with fewer children (14.7%-16.7%) and adolescents (13.2%-14.7%) having at least one concomitant-use event. Prevalence rates remained largely similar across the years for this subpopulation, with a few exceptions: in children, the prevalence of guanfacine IR use increased over time and use of clonidine IR, SSRIs, and AAPs decreased over time, whereas in adolescents, the prevalence of guanfacine XR use increased and use of AAPs decreased over time.

Among children with noncomorbid ADHD, guanfacine XR was the most used concomitant psychotropic medication every year, with a notable increase during 2012. In adolescents, although the overall percentage of adolescents using at least one drug to augment their stimulant regimen rose between 2011 and 2014, use by the subpopulation of adolescents with noncomorbid ADHD stayed relatively consistent. Among these adolescents, SSRIs remained the most used concomitant therapy despite decreasing prevalence for each year analyzed.

It is possible that some of the children and adolescents, considered in this analysis to have noncomorbid ADHD, may have had mental health conditions that were not recorded in the MarketScan database, perhaps due to the stigma attached to a psychiatric diagnosis. Furthermore, results from this subpopulation of children and adolescents with noncomorbid ADHD will not be generalizable to the entire ADHD population, many of whom are characterized as having a heavy comorbidity burden (Thomas, 2013). For these reasons, the true prevalence rate of concomitant psychotropic drug use among children and adolescents with ADHD most likely falls between the estimate for the subpopulation with noncomorbid ADHD and the estimate for the overall population.

This study was conducted using administrative claims data, which are inherently associated with certain limitations. Concomitant psychotropic medication use was defined based on a period of overlapping prescription fills rather than the clinician’s intention to treat adjunctively. This approach allowed real utilization to be captured (i.e., whether the children and adolescents actually filled the prescriptions) but did not provide insight into the specific reasons for prescribing or the intended frequency of adjunctive therapy in ADHD. In addition, these results are only generalizable to the commercially insured population in the United States, which may not be representative of all U.S. children and adolescents, particularly, those with public insurance.

## Conclusion

Concomitant use of psychotropic medication with stimulants was prescribed for around a quarter of children and adolescents in the United States between 2011 and 2014 despite the fact that most medication classes studied were not approved by the FDA for concomitant use with psychostimulants during that time. Such concomitant use of psychotropic medication with stimulants was observed in both children and adolescents in the overall population, and in the sensitivity analysis among a subpopulation that did not have any psychiatric or neurologic comorbidities. These results underscore the need for adjunctive options for use during stimulant treatment of ADHD. In the management of ADHD among children and adolescents, clinicians should evaluate patients on a case-by-case basis, taking into account their unique characteristics and the defined risk–benefit profile for the different treatment options. The risk–benefit profiles of many medications used in combination with stimulants in practice are not well described (Bussing & Winterstein, 2012). However, two nonstimulants (guanfacine XR and clonidine XR) have been shown to be effective and well tolerated for adjunctive use with stimulants in children and adolescents with ADHD (Childress, 2012; Ming et al., 2011) and have been approved by the FDA as adjunctive therapy with stimulants. Further research is needed to evaluate the efficacy, safety, and economic impact of using psychotropic medications concomitantly with other ADHD medication.

## Appendix A

**Table:**

Comorbidity	ICD-9 code
Psychiatric and other neurological disorders for which atypical antipsychotics are indicated	
Bipolar disorder	296.4, 296.5, 296.6, 296.7, 296.8
Dementia	290, 294.1
Mania	296.0, 296.1
Other psychotic disorders	293.81, 293.82, 297.1, 297.3, 298.8, 298.9
Schizophrenia	295
Tics	307.2
Psychiatric (and other neurological) comorbidities of ADHD	
Adjustment reaction	309.xx
Anxiety disorder	293.84, 300.0x, 300.2x, 313.0x
Conduct disorder	312.xx
Depression	296.2x, 296.3x, 311.xx, 300.4x
Epilepsy	345.xx
Insomnia	307.41, 307.42, 327.0x, 780.51, 780.52
Learning disability	315.xx
Obsessive-compulsive disorder	300.3x
Oppositional defiant disorder	313.81
Substance abuse	291.xx, 292.xx, 303.xx, 304.xx, 305.xx
Pervasive developmental disorder	299.x
Other neurological disorders	320.xx-337.xx, 340.xx-349.xx excluding 345.xx

*Source.* Adapted from Centers for Disease Control and Prevention (2017).

*Note*. ICD-9 = The International Conference for the Ninth Revision of the International Classification of Diseases.
